# Clinical evaluation of medical and surgical complete responses in metastatic renal cell carcinoma treated with immune checkpoint inhibitor combination therapy

**DOI:** 10.1007/s10147-026-02981-9

**Published:** 2026-02-06

**Authors:** Kazuhiko Yoshida, Tsunenori Kondo, Junpei Iizuka, Yuki Kobari, Hiroki Ishihara, Hironori Fukuda, Hiroaki Shimmura, Yasunobu Hashimoto, Hiroshi Kobayashi, Hideki Ishida, Toshio Takagi

**Affiliations:** 1 Department of Urology, Tokyo Women’s Medical University, 8-1, Kawada-cho, Shinjuku-ku, Tokyo, 162-8666 Japan; 2 Department of Urology, Adachi Medical Center, Tokyo Women’s Medical University, 4-33-1, Kouhoku, Adachi-ku, Tokyo Japan; 3 Department of Urology, Joban Hospital, Uenodai 57, Joban Kamiyunagayamachi, Iwaki, Fukushima Japan; 4 Department of Urology, Saiseikai Kawaguchi General Hospital, 5-11-5 Nishikawaguchi, Kawaguchi, Saitama Japan; 5 Department of Urology, Saiseikai Kazo Hospital, 1680 Kamitakayanagi, Kazo, Saitama Japan

**Keywords:** Metastatic renal cell carcinoma, Immune checkpoint inhibitor-based combination, Surgical complete response, Medical complete response, Multidisciplinary treatment

## Abstract

**Background:**

Achieving a complete response (CR) with immune checkpoint inhibitor (ICI)-based combination therapy is important in metastatic renal cell carcinoma (mRCC) systemic treatment. Surgical intervention for residual localized disease after ICI therapy may contribute to complete disease eradication and improved outcomes.

**Methods:**

We retrospectively evaluated the clinical significance of medical CR (complete radiologic disappearance of all target lesions with ICI therapy) and surgical CR (radiographic CR after local surgery following ICI-based therapy) for patients with mRCC treated with ICI-based combination therapy. Patients were categorized into the IOIO (dual ICI therapy) and IOTKI (ICI + tyrosine kinase inhibitor therapy) groups.

**Results:**

Of the 250 study patients, 107 and 143 received IOIO and IOTKI, respectively. The overall medical objective response and medical CR rates were 55.6% and 10.4%, respectively. Surgical CR and medical CR were achieved in 16.4% and 26.8% of individuals, respectively. Patients who achieved both medical CR and surgical CR experienced similarly favorable progression-free survival and overall survival (OS). Among those who achieved either surgical CR or medical CR, OS was longer in the IOIO group; however, no other significant intergroup differences were observed. Patients with primary tumors exhibited higher medical CR rates. No significant differences in treatment-related adverse events, treatment discontinuation, or steroid use between the medical CR and medical non-CR groups were observed.

**Conclusion:**

Approximately 25% of patients with mRCC achieved medical CR with ICI-based combination therapy. Treatment efficacy was comparable between the two regimen groups. A multidisciplinary strategy may lead to complete disease eradication for select patients.

**Supplementary Information:**

The online version contains supplementary material available at 10.1007/s10147-026-02981-9.

## Introduction

Systemic treatment for metastatic renal cell carcinoma (mRCC) has evolved from cytokine therapy to tyrosine kinase inhibitors (TKIs) and, more recently, immune checkpoint inhibitor (ICI)-based combination therapies. With cytokine treatment, high-dose interleukin-2 treatment resulted in spontaneous tumor regression in a limited patient subset; nonetheless, the complete response (CR) rate remained low (approximately 3%) [1–3]. With TKI treatment, improvements in overall survival (OS) and progression-free survival (PFS) were achieved; however, the CR rate was generally less than 5% [4, 5].

Recently, the advent of ICI treatment has resulted in remarkably improved outcomes. Several studies have reported not only enhanced OS and PFS but also increased CR rates (10–16%) [6–10]. Moreover, the depth of treatment responses, such as CR or partial response (PR), has been associated with durable benefits and the potential for long-term disease control. Kimura et al. reported that the complete disappearance of cancer is the outcome most anticipated by patients with mRCC; additionally, CR is crucial to survival, quality of life, and psychological well-being [11]. Accordingly, medical CR has become a crucial therapeutic goal in mRCC systemic therapy.

Systemic TKI therapy has been recommended as initial treatment for mRCC after evidence of noninferiority of TKI alone compared with TKI plus upfront cytoreductive nephrectomy (uCN) as well as evidence of the potential benefit of deferred cytoreductive nephrectomy (dCN) compared with uCN were confirmed, and dCN was considered as a subsequent option [4, 5]. Similarly, recent studies have suggested that dCN after ICI-based combination therapy may also be beneficial [12, 13]. In addition, the clinical utility of complete metastasectomy after primary tumor resection in mRCC is well recognized, and surgical CR is an important component of this multidisciplinary treatment strategy [14–18]. Moinard-Butot et al. recently emphasized the value of combining ICI-based therapies with local treatments, such as dCN, metastasectomy, or stereotactic radiotherapy, for residual disease [19]. However, despite these advances in treatment, the real-world frequency, determinants, and prognostic implications of medical CR and surgical CR with current ICI treatments are insufficiently characterized. Since medical CR with ICI combination therapy alone is limited, we hypothesized that a subset of patients with stable disease or PR after systemic therapy may achieve complete eradication of residual disease with surgical interventions such as deferred cytoreductive nephrectomy (dCN) and/or metastasectomy. Therefore, we defined a composite endpoint termed “treatment CR,” which encompasses both medical CR and surgical CR, that represents achievable complete disease eradication with a multidisciplinary strategy. In this study, the primary exploratory endpoints were the frequency and prognosis of achievable complete disease control with a multidisciplinary strategy that combines ICI-based systemic therapy and local treatments. The secondary exploratory endpoints were the clinical characteristics of patients with medical CR compared with those of patients with surgical CR and differences in the achievement of treatment CR and associated clinical outcomes of patients treated with dual ICI therapy (IOIO) and those of patients treated with ICI plus TKI therapy (IOTKI).

## Patients and methods

### Study design

This multicenter, retrospective, observational study was approved by the Institutional Review Board of Tokyo Women’s Medical University (approval no. 2021–0061) and conducted in accordance with ethical standards of the 1964 Declaration of Helsinki and its later amendments. Because of the retrospective design of this study, the requirement for informed consent was waived.

### Patients and data collection

This study was conducted at Tokyo Women’s Medical University and four affiliated institutions (Tokyo Women’s Medical University Adachi Medical Center, Saiseikai Kawaguchi General Hospital, Saiseikai Kazo Hospital, and Jyoban Hospital). Between January 2018 and December 2024, 278 patients with advanced or mRCC received ICI-based combination therapy as first-line systemic treatment, which included nivolumab plus ipilimumab, pembrolizumab plus axitinib, avelumab plus axitinib, nivolumab plus cabozantinib, and pembrolizumab plus lenvatinib. After excluding 28 patients because of incomplete clinical data, 250 patients were categorized into the IOIO group and IOTKI group and included in the final analysis.

Clinical and laboratory information was retrospectively collected from medical records and electronic databases. Detailed clinical variables of the patients are listed in Table 1.

**Table 1:** Clinical characteristics of patients with mRCC treated with ICI-based combination treatments

		Total	IOIO	IOTKI	*p*-value
		n = 250	n = 107	n = 143	
*Patient characteristics*					
Sex, n (%)	Male	187 (74.8)	79 (73.8)	108 (75.5)	0.7604
Age, years, mean ± SD		65.6 ± 11.9	63.2 ± 12.0	67.4 ± 11.4	0.0055
ECOG-PS, n (%)	0	149 (59.6)	66 (61.7)	83 (58.0)	0.5617
	≥ 1	101 (40.4)	41 (38.3)	60 (42.0)	
Body mass index, kg/m^2^, mean ± SD	23.6 ± 5.7	23.3 ± 4.3	23.9 ± 6.6	0.3787	
Kidney surgery, n (%)	Yes	169 (67.6)	73 (68.2)	96 (67.1)	0.8552
IMDC risk classification, n (%)	Favorable	31 (12.4)		31 (21.7)	< 0.0001
	Intermediate	146 (58.4)	70 (65.4)	76 (53.2)	
	Poor	70 (28.0)	36 (33.6)	34 (23.8)	
	Unknown	3 (1.2)	1 (0.9)	2 (1.4)	
*Tumor factors*					
Histopathology, n (%)	Clear cell carcinoma	180 (72.0)	79 (73.8)	101 (70.6)	0.3259
	Nonclear cell carcinoma	43 (17.2)	20 (18.7)	23 (16.1)	
	Unknown	27 (10.8)	8 (7.5)	19 (13.3)	
	Sarcomatoid feature	21 (8.4)	8 (7.5)	13 (9.1)	0.2836
*ICI combination therapy*					
Nivolumab plus ipilimumab, n (%)		107 (42.8)	107 (100)	–	–
Pembrolizumab plus axitinib, n (%)		26 (10.4)	–	26 (18.2)	
Avelumab plus axitinib, n (%)		18 (7.2)	–	18 (12.6)	
Nivolumab plus cabozantinib, n (%)		54 (21.6)	–	54 (37.8)	
Pembrolizumab plus lenvatinib, n (%)		45 (18.0)	–	45 (31.5)	
*Post-treatment outcomes*					
Follow-up duration, months, mean ± SD		22.5 ± 16.9	28.4 ± 18.8	18.0 ± 14.0	< 0.0001

*ECOG-PS*, eastern cooperative oncology group performance status; *ICI*, immune checkpoint inhibitor; *IMDC*, international metastatic renal cell carcinoma database consortium; *IOIO*, dual immune checkpoint inhibitor therapy; *IOTKI*, immune checkpoint inhibitor plus tyrosine kinase inhibitor therapy; *mRCC*, metastatic renal cell carcinoma; *SD*, standard deviation

We applied a previously reported protocol of ICI-based combination therapy as the first-line mRCC treatment [20–24]. Treatment was selected through discussions between the attending physicians and patients at each institution, without standardized criteria, and based on a clear consensus. When selecting the optimal ICI-based combination therapy, we considered patient variables, tumor factors, treatment efficacy, and other individual characteristics. Metastasectomy or dCN of the remaining tumor was performed when nearly all disease sites, except for resectable lesions, were considered controlled by systemic therapy. Disease controlled with systemic therapy and disease compatible with CR with systemic therapy were assessed radiologically. A small residual lesion that was considered controlled was classified as a “nonviable scar tissue” when the maximal diameter was 5 mm or less, no contrast enhancement was observed on computed tomography or magnetic resonance imaging, it remained stable or reduced in size during a period of at least 3 months, and it exhibited radiologic features considered clinically consistent with nonviable fibrotic tissue. Surgical decisions were based on clinical judgment and made at the discretion of the treating physician. No standardized protocols were available for patient selection or surgical timing. Treatment was discontinued if surgical CR occurred. Medical treatment was discontinued after surgery based on the judgment of the treating physician or preference of the patient because no standardized institutional protocol existed.

### Definitions and outcomes

CR was assessed according to the Response Evaluation Criteria in Solid Tumors version 1.1. Medical CR was defined as the complete disappearance of all target lesions achieved with ICI-based combination therapy according to a radiologic assessment. Surgical CR was defined as complete disease disappearance achieved after local surgical treatment, such as dCN or metastasectomy, following ICI combination therapy according to a radiologic assessment. Treatment CR was defined as both medical CR and surgical CR and represented achievable complete disease eradication using a multidisciplinary strategy that combined systemic therapy and local surgical intervention. The clinical significance of CR, whether medical or surgical, was retrospectively evaluated. The objective response rate (ORR) was identified as the sum of CRs and PRs. PFS, recurrence-free survival (RFS), and OS were defined as the time from initiation of ICI-based combination therapy to the time of radiographic disease progression, radiographic disease recurrence, or death from any cause, respectively. Radiologic response evaluations were conducted using contrast-enhanced computed tomography of the chest, abdomen, and pelvis every 8–12 weeks, depending on the clinical status of the patient. Additional imaging modalities were used based on clinical judgment. Treatment continued until radiographic or clinical progression occurred or until intolerable adverse events occurred.

### Safety evaluation

Safety profiles were assessed based on the treatment-related adverse event (TRAE) incidence, treatment interruption or discontinuation rates (defined as interruption or discontinuation of both agents comprising combination therapy), and high-dose corticosteroid use. TRAEs were graded according to the Common Terminology Criteria for Adverse Events version 5.0 [25]. Treatment interruption and discontinuation were defined as cessation of any ICI-based combination component because of toxicity or patient preference. However, treatment interruption and discontinuation could not be strictly distinguished in this study. ICIs were administered at fixed doses without modification, whereas the TKI dose was adjustable based on toxicity and the clinical status.

### Statistical analysis

Comparisons between treatment groups were performed using the Chi-square or Fisher’s exact tests for categorical variables and the Mann–Whitney U test for continuous variables, as appropriate. Kaplan–Meier curves were generated for PFS, RFS, and OS. Differences between groups were assessed using the log-rank test. In addition, *p* < 0.05 was considered statistically significant. All statistical analyses were conducted using JMP Pro version 17.0.0 (SAS Institute, Cary, NC, USA).

## Results

### Patient characteristics

Clinical and demographic characteristics of patients are summarized in Table 1. A total of 250 patients (187 [74.8%] male and 63 [25.2%] female patients) with a mean age of 65.6 ± 11.9 years were included in this study; 107 and 143 patients comprised the IOIO and IOTKI groups, respectively. Patients in the IOIO group were younger than those in the IOTKI group (*p* = 0.0055). Follow-up of the IOIO group was significantly longer than that of the IOTKI group (*p* < 0.0001). No significant differences in other baseline characteristics, except for the International Metastatic Renal Cell Carcinoma Database Consortium risk classification, were observed between these groups.

### Medical CR

**Fig. 1:**
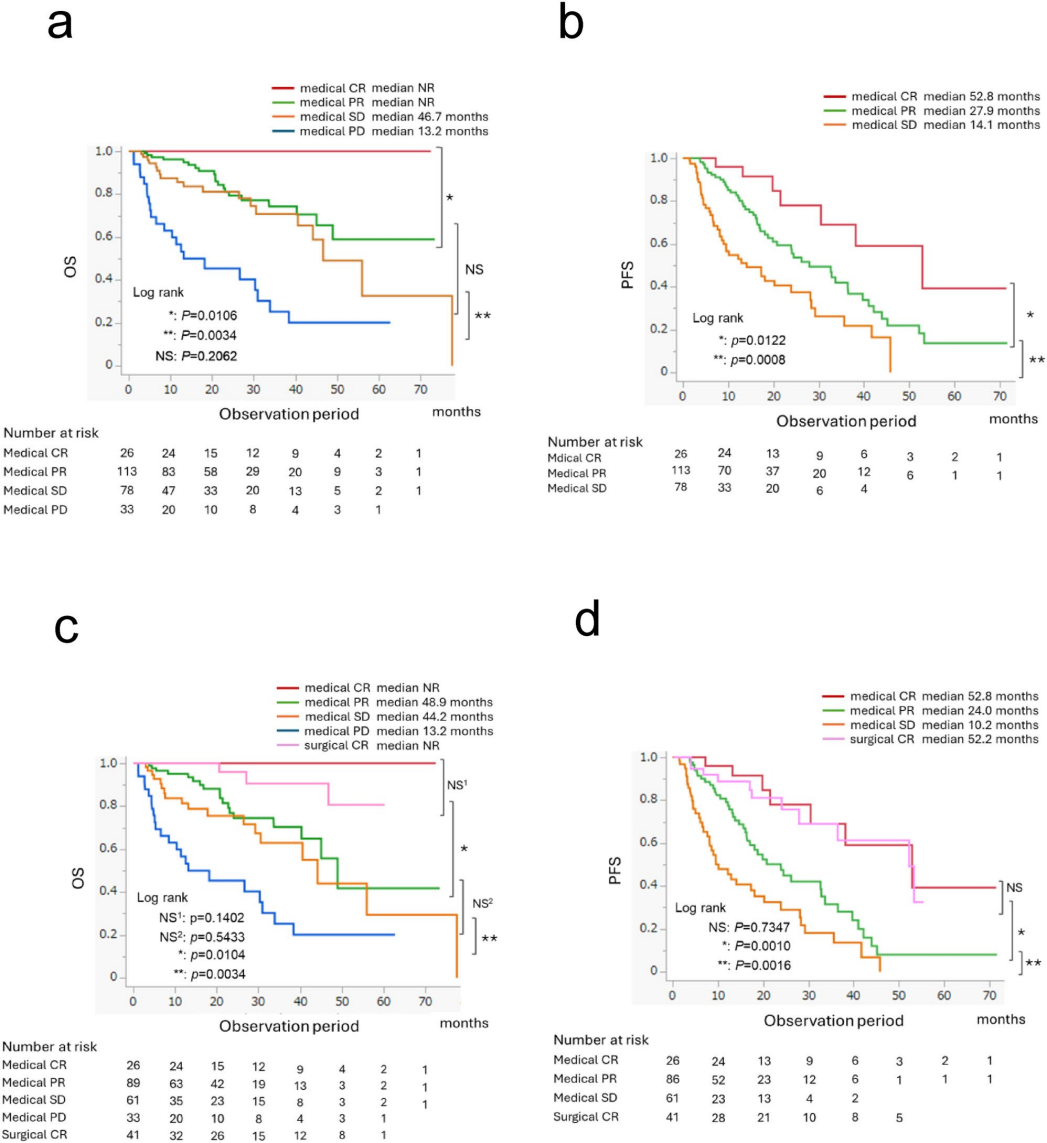
Overall survival and progression-free survival of the entire cohort according to the treatment response. **a** Overall survival of the entire cohort. **b** Progression-free survival in the entire cohort. **c** Overall survival of patients who achieved surgical complete response. **d** Progression-free survival of patients who achieved surgical complete response. CR, complete response; OS, overall survival; PD, progressive disease; PFS, progression-free survival; PR, partial response; SD, stable disease; NR, not reached; NS, not significant

**Fig. 2:**
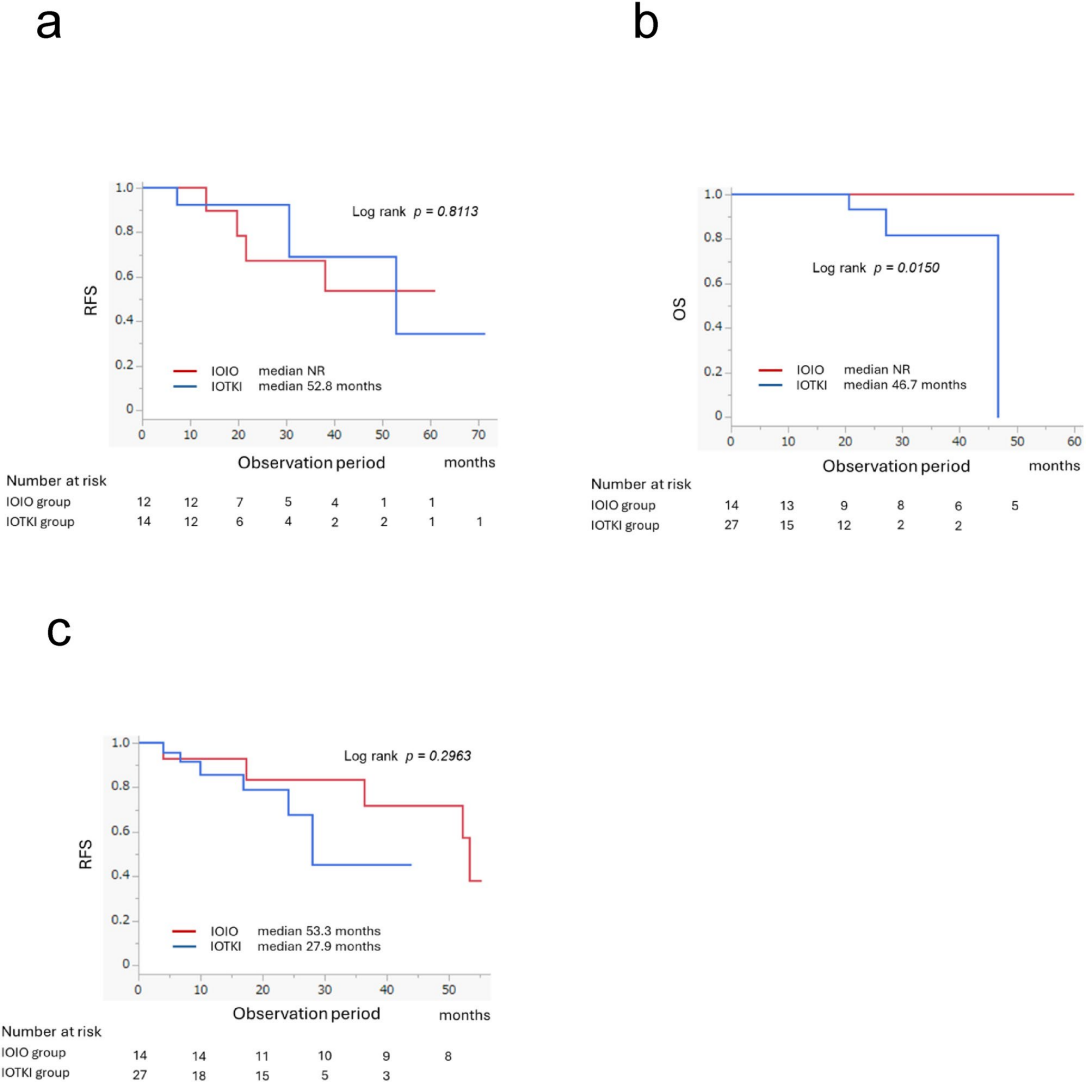
Comparison of treatment outcomes of patients in the IOIO and IOTKI groups who achieved medical complete response or surgical complete response. **a** Recurrence-free survival of patients with medical complete response: IOIO vs. IOTKI. **b** Overall survival of patients with surgical complete response: IOIO vs. IOTKI. **c** Recurrence-free survival of patients with surgical complete response: IOIO vs. IOTKI. IOIO, dual immune checkpoint inhibitor therapy; IOTKI, immune checkpoint inhibitor plus tyrosine kinase inhibitor therapy; NR, not reached; OS, overall survival; RFS, recurrence-free survival

ICI-based combination therapy treatment responses are shown in Table 2. The overall mean tumor shrinkage rate was − 33.0% ± 46.9%; no statistically significant difference between the IOIO and IOTKI groups was observed (*p* = 0.1572). The progressive disease rate in the IOTKI group was significantly lower than that in the IOIO group (*p* < 0.0001). Patients with medical CR (10.4%) experienced favorable outcomes, and none died during follow-up (median PFS, 52.8 months) (Fig. 1a, b). Among patients with medical CR, no OS events occurred in the IOIO group or IOTKI group, and no significant difference in their PFS was observed (Fig. 2a).

**Table 2:** Efficacy of ICI-based combination therapy and details of surgical interventions for residual tumors following systemic therapy in mRCC

	Total	IOIO	IOTKI	*p*-value
	n = 250	n = 107	n = 143	
*Tumor*				
Tumor size before treatment, mm, mean ± SD	71.1 ± 56.6	77.0 ± 58.5	66.8 ± 54.9	0.1642
Tumor size at the time of best response, mm, mean ± SD	50.4 ± 53.0	56.3 ± 59.6	46.1 ± 47.4	0.1347
Reduction ratio of the tumor at the time of best response, %, mean ± SD	− 33.0 ± 46.9	− 28.0 ± 52.2	− 36.8 ± 42.4	0.1572
*Response rate*				
Medical ORR, n (%)	139 (55.6)	51 (47.7)	88 (61.6)	0.2206
Medical CR, n (%)	26 (10.4)	12 (11.2)	14 (9.8)	0.7150
Medical PR, n (%)	113 (45.2)	39 (36.4)	74 (51.7)	0.0162
Medical stable disease, n (%)	78 (31.2)	26 (24.3)	52 (36.4)	0.0520
Medical PD, n (%)	33 (13.2)	30 (28.0)	3 (2.1)	< 0.0001
Surgical CR, n (%)	41 (16.4)	14 (13.1)	27 (18.8)	0.4400
Treatment (medical + surgical) CR, n (%)	67 (26.8)	26 (24.3)	41 (28.7)	0.2723
*Surgery following systemic therapy*				
Deferred CN, n (%)	35 (14.0)	12 (11.2)	23 (16.1)	0.3679
Metastasectomy, n (%)	13 (5.2)	4 (3.7)	9 (6.3)	0.5285
Lung, n (%)	7 (2.8)	3 (2.8)	4 (2.8)	
Liver, n (%)	2 (0.8)	–	2 (1.4)	
Adrenal gland, n (%)	1 (0.4)	–	1 (0.7)	
Contralateral kidney, n (%)	1 (0.4)	–	1 (0.7)	
Lymph node, n (%)	1 (0.4)	–	1 (0.4)	
Retroperitoneal, n (%)	1 (0.4)	1 (0.9)	–	

*CR*, complete response; *ICI*, immune checkpoint inhibitor; *IOIO*, dual immune checkpoint inhibitor therapy; *IOTKI*, immune checkpoint inhibitor plus tyrosine kinase inhibitor therapy; *mRCC*, metastatic renal cell carcinoma; *ORR*, objective response rate; *PD*, progressive disease; *PR*, partial response; *SD*, standard deviation

### Surgical CR

A total of 41 patients underwent surgery and achieved surgical CR following systemic therapy; 35 and 13 patients underwent dCN and metastasectomy, respectively. Among these patients, seven underwent dCN and metastasectomy, and 14 and 27 were in the IOIO and IOTKI groups, respectively (Table 2). Patients with surgical CR experienced favorable outcomes; the median OS was not reached (*p* = 0.1402) and the median PFS was 52.2 months (*p* = 0.7347) (Fig. 1c, d). A comparison of surgical CR in the IOIO and IOTKI groups showed that OS was significantly better in the IOIO group (*p* = 0.0150) (Fig. 2b); however, no significant difference in RFS was observed (*p* = 0.2963) (Fig. 2c).

### Treatment CR

**Fig. 3:**
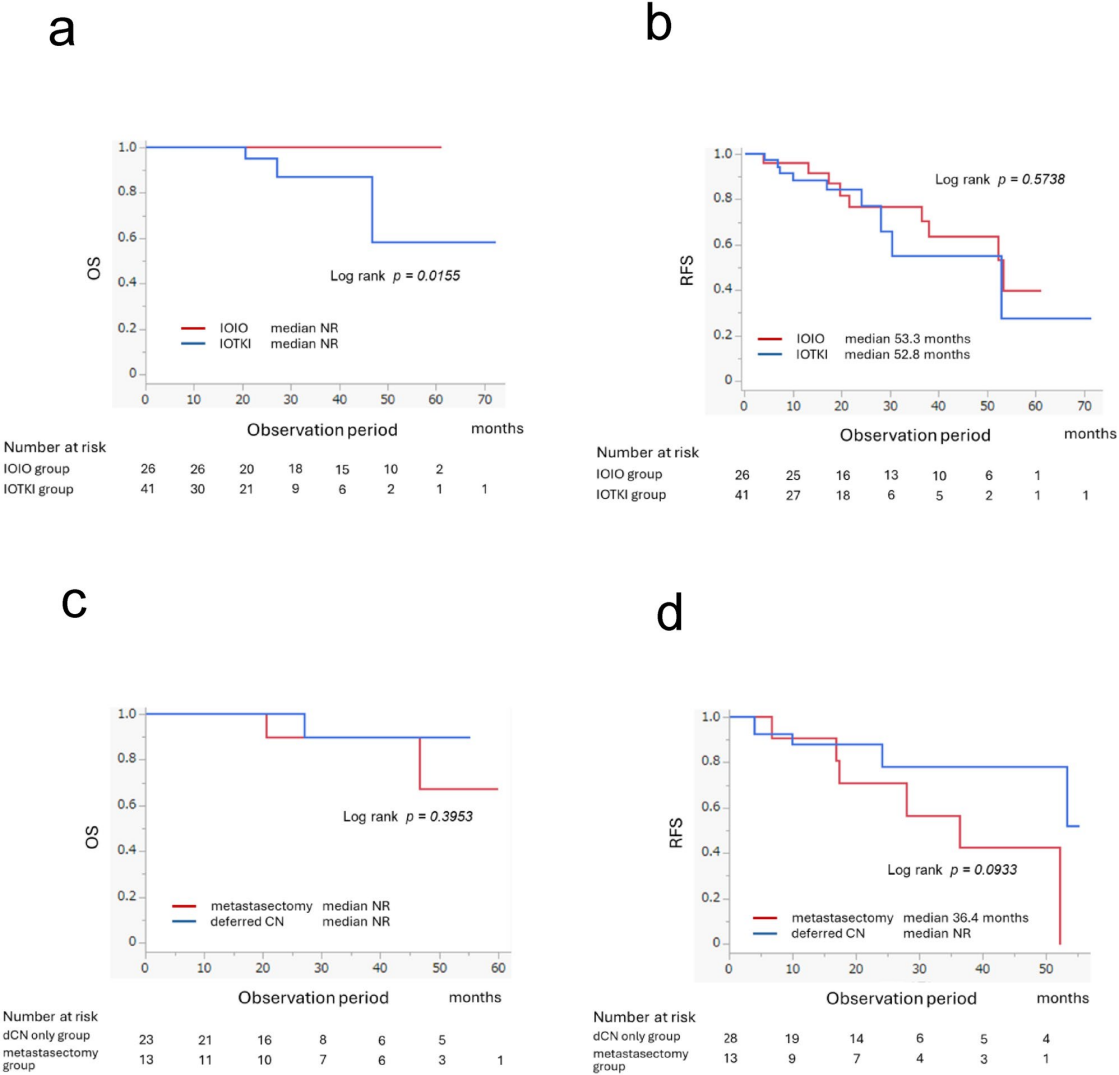
Survival outcomes of patients who achieved treatment complete response and comparison between deferred cytoreductive nephrectomy and metastasectomy among patients who achieved surgical complete response. **a** Overall survival of patients with treatment complete response: IOIO vs. IOTKI. **b** Recurrence-free survival of patients with treatment complete response: IOIO vs. IOTKI. **c** Overall survival: dCN only vs. metastasectomy. **d** Recurrence-free survival: dCN only vs. metastasectomy. dCN, deferred cytoreductive nephrectomy; IOIO, dual immune checkpoint inhibitor therapy; IOTKI, immune checkpoint inhibitor plus tyrosine kinase inhibitor therapy; NR, not reached; OS, overall survival; RFS, recurrence-free survival

**Fig. 4:**
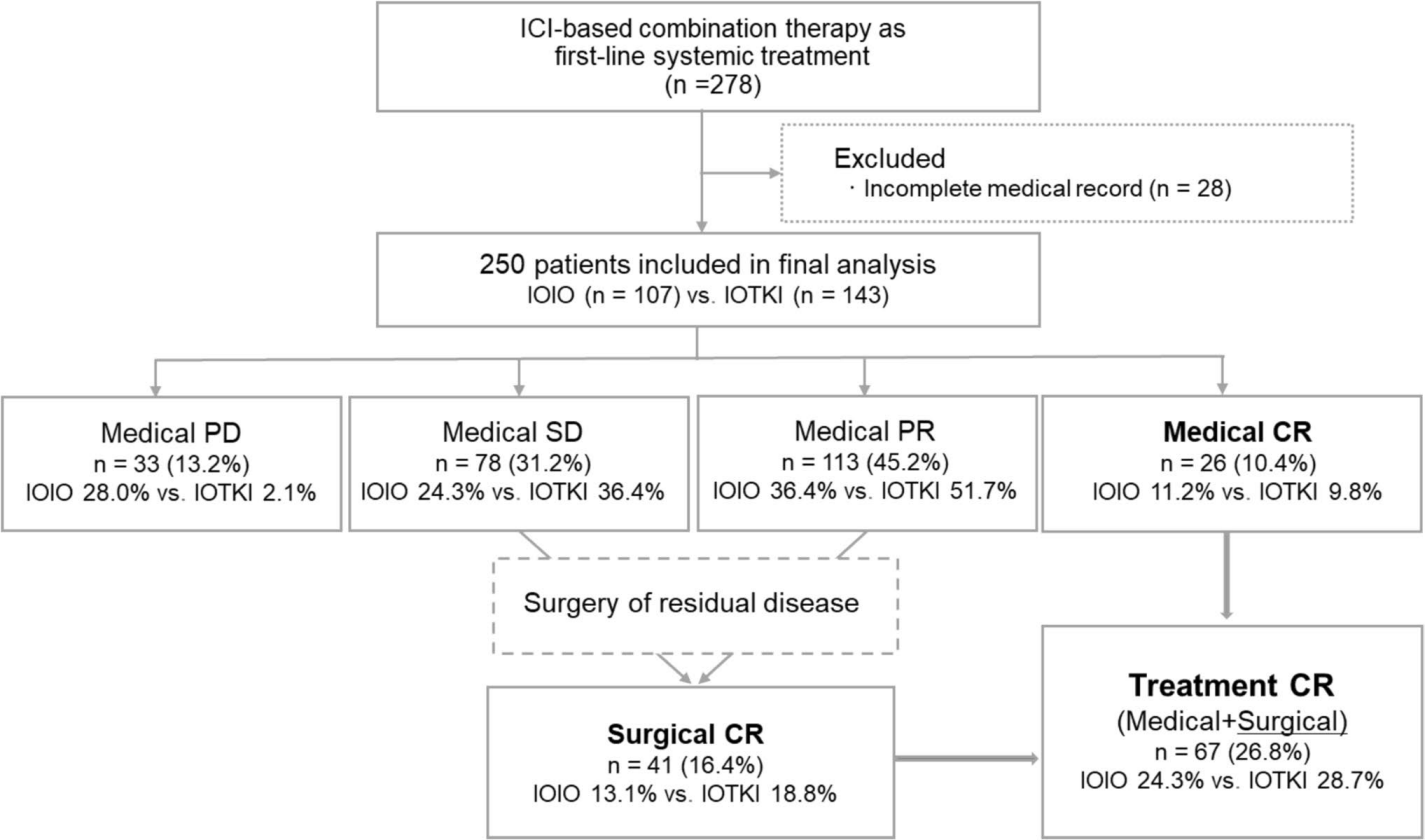
Patient selection and treatment response flowchart. Patients in the final analysis were categorized into two treatment groups: IOIO (ipilimumab plus nivolumab) and IOTKI (ICI plus tyrosine kinase inhibitors) groups. Treatment response was evaluated according to RECIST v.1.1, and the proportions of medical CR, PR, SD, and PD are shown. In some patients, surgical CR was achieved through deferred cytoreductive nephrectomy or metastasectomy. The treatment CR rate reflects the combined proportion of patients who achieved either medical CR or surgical CR. CR, complete response; ICI, immune checkpoint inhibitor; PD, progressive disease; PR, partial response; RECIST, Response Evaluation Criteria in Solid Tumors; SD, stable disease

The treatment CR rate, which included medical CR and surgical CR, was 26.8%. For patients with treatment CR, the median OS was not reached and the median RFS was 52.8 months. A comparison of patients in the IOIO and IOTKI groups with treatment CR showed that OS was significantly better in the IOIO group (*p* = 0.0155); however, RFS did not significantly differ (*p* = 0.5738) (Fig. 3a, b). No significant differences in OS or RFS were observed between patients who underwent dCN and those who underwent metastasectomy (Fig. 3c, d). Figure 4 shows the baseline characteristics and treatment outcomes of patients with advanced mRCC who received first-line ICI-based combination therapy.

### Response according to the primary tumor status and histopathological subtype

The ORR according to the primary tumor status is shown in Table 3. Patients with primary tumors did not achieve medical CR; however, surgical CR and treatment CR occurred more frequently in these patients. Regardless of the primary tumor status, no significant differences in CR rates were observed between the IOIO and IOTKI groups. The ORR according to the histopathological subtype is summarized in Online Resource 1; however, it should be interpreted as exploratory because of the small number of patients with nonclear cell mRCC who achieved CR.

**Table 3:** Efficacy of ICI-based combination therapy for mRCC: comparison of outcomes and treatment CR (medical CR and surgical CR) rates between patients with and without primary tumors

	Primary tumor presence			Primary tumor absence		
	n = 81			n = 169		
	IOIO	IOTKI	*p*-value	IOIO	IOTKI	*p*-value
	n = 34	n = 47		n = 73	n = 96	
*ORR*						
Medical ORR, n (%)	17 (50.0)	19 (40.4)	0.3921	34 (46.6)	69 (71.9)	0.0008
Medical CR, n (%)	0	0	–	12 (16.4)	14 (14.6)	0.7406
Medical PR, n (%)	17 (50.0)	19 (40.4)	0.3921	22 (30.1)	55 (57.3)	0.0004
Medical stable disease, n (%)	4 (11.8)	28 (59.6)	< 0.0001	22 (30.1)	24 (25.0)	0.4573
Medical PD, n (%)	13 (38.2)	0	< 0.0001	17 (23.3)	3 (3.1)	< 0.0001
Surgical CR, n (%)	11 (32.4)	21 (44.7)	0.2627	3 (4.1)	6 (6.3)	0.5393
Treatment (medical and surgical) CR, n (%)	11 (32.4)	21 (44.7)	0.2627	15 (20.6)	20 (20.8)	0.9638

*CR*, complete response; *ICI*, immune checkpoint inhibitor; *IOIO*, dual immune checkpoint inhibitor therapy; *IOTKI*, immune checkpoint inhibitor plus tyrosine kinase inhibitor therapy; *mRCC*, metastatic renal cell carcinoma; *ORR*, objective response rate; *PD*, progressive disease; *PR*, partial response

### Safety

Safety outcomes are shown in Table 4. TRAEs of any grade and grade ≥ 3 TRAEs were not statistically significantly different between treatment groups (*p* = 0.1126 and *p* = 0.6980, respectively). Treatment interruption and discontinuation rates attributable to TRAEs were not significantly different between the treatment CR and treatment non-CR groups (*p* = 0.0698). High-dose steroid administration was not significantly different between the treatment CR and treatment non-CR groups (*p* = 0.6137).

**Table 4:** Safety of ICI-based therapy for mRCC: comparison of outcomes between patients with and without treatment CR

	Total	Treatment CR	Non-treatment CR	*p*-value
	n = 250	n = 67	n = 183	
*TRAE*				
TRAE; all grades, n (%)	222 (88.8)	63 (94.0)	159 (86.9)	0.1126
TRAE ≥ grade 3, n (%)	133 (53.2)	37 (55.2)	96 (52.5)	0.6980
Interruption, n (%)	55 (22.0)	20 (29.9)	35 (19.1)	0.0698
Sustained high-dose steroid, n (%)	87 (34.8)	25 (37.3)	62 (33.9)	0.6137

*CR*, complete response; *ICI*, immune checkpoint inhibitor; *mRCC*, metastatic renal cell carcinoma; *TRAE*, treatment-related adverse event

## Discussion

This is the first retrospective multicenter study to investigate the frequency and outcomes of medical CR and surgical CR among patients with mRCC and compare ICI-based therapies (IOIO vs. IOTKI) using a composite endpoint for complete disease eradication.

With the advent of ICI-based combination therapies, medical CR and long-term survival have become increasingly realistic goals in mRCC treatment [6–10]. These results highlight a significant improvement over cytokine-based or TKI monotherapy and the clinical impact of ICI-based combination therapy [1–5]. Nevertheless, whether ICI-based combination therapy should be combined with surgery is unclear. Recent research suggested that dCN following ICI-based combination therapy may improve outcomes [12, 13, 26, 27], and high medical PR rates suggest that tumor shrinkage may increase the number of patients eligible for surgical intervention. This study found that patients without primary tumors more commonly achieved medical CR. Although medical CR was not observed in patients with primary tumors, some achieved pathological CR after dCN. Notably, 39.5% of patients with primary tumors achieved surgical CR after systemic therapy combined with local interventions. Therefore, patients with mRCC and primary tumors who receive ICI-based regimens may derive a survival benefit from nephrectomy. Subsequently, several prospective trials are currently underway, and their results may clarify the clinical utility of dCN [28, 29].

Furthermore, metastasectomy may be clinically beneficial, particularly for cases involving a limited number of metastatic or progressing lesions [15–18]. Nonetheless, few studies have addressed the effectiveness of combining ICI therapy with surgical removal of localized residual metastatic lesions in other organs. Moinard-Butot et al. reported that 80 patients with clear cell mRCC treated with ICI-based regimens had an ORR of 56%, and that 10% of participants achieved CR through systemic therapy alone and an additional 14% achieved CR following local treatments such as dCN, metastasectomy, radiotherapy, or ablation, resulting in a treatment CR rate of 24% [19]. Most of these patients maintained their disease-free status during follow-up. Our study consistently demonstrated that surgical intervention following ICI-based combination therapy resulted in surgical CR in 16.4% of patients and a treatment CR rate of 26.8%. Those with surgical CR had a median PFS of 52.2 months, which was similar to that of the medical CR group (median PFS, 52.8 months). Therefore, long-term tumor control may be achieved not only with systemic therapy but also with additional local interventions for patients who do not achieve medical CR. Although deep responses to systemic therapy are considered predictors of favorable outcomes, these results indicate that surgical CR may also contribute to durable disease control [30], thus underscoring the importance of a multidisciplinary treatment strategy involving ICIs. Select patients with residual but resectable disease after systemic treatment may achieve complete disease eradication with surgery. Although no significant differences in medical, surgical, or treatment CR were observed between treatment groups, the progressive disease rate of the IOTKI group was significantly lower than that of the IOIO group. Conversely, the IOIO group had more durable responses and longer OS among those in the surgical CR and treatment CR subgroups despite similar RFS. Thus, both regimens have clinical value, and treatment selection should be individualized based on patient characteristics, tumor biology, and drug profiles.

Importantly, no significant differences in TRAEs, treatment discontinuation, or steroid use were observed between patients who did and did not achieve treatment CR, indicating that treatment CR can be achieved without compromising safety. The treatment discontinuation rate attributable to TRAEs in the treatment CR group was higher than that in the non-CR group, indicating that treatment was discontinued after CR and considering the patient’s condition at that time and the clinical judgment of the treating physician. Evidence in the field of melanoma supports the feasibility of discontinuing ICI therapy following CR [31, 32]. Previous reports have shown that > 85% of patients with melanoma who discontinued ICI therapy after CR maintained a durable response [33]. Nevertheless, evidence that supports treatment discontinuation after CR with ICI-based combination therapy for mRCC is scarce. Prospective studies are warranted to determine the optimal timing and predictors of treatment discontinuation for patients with treatment CR. A multivariate analysis did not identify significant predictive factors for medical CR in our study. Future research should identify novel biomarkers for improved treatment responses in mRCC.

Our study had some limitations. Since it was a retrospective analysis, selection bias was possible, particularly because treatment decisions were made at the discretion of the physician. Patients in the IOIO group were younger than those in the IOTKI group; this age difference possibly reflected TRAEs. Choosing between IOIO and IOTKI may have been influenced by International Metastatic Renal Cell Carcinoma Database Consortium risk classification. Differences in follow-up duration among groups and the absence of standardized criteria for local interventions across institutions may have further affected outcomes. In addition, radiologic evaluations were performed by the treating physicians at each participating institution without central or blinded review, and interinstitutional variability in image interpretation may have influenced the CR determination, particularly for cases with small residual lesions. The impact of treatment discontinuation, whether because of TRAEs or surgery completion, on RFS was not evaluated because decisions regarding systemic therapy discontinuation after surgical intervention were left to the discretion of the treating physician, thereby introducing further variability. Our study did not include cases treated with stereotactic body radiation or ablation therapies. However, the efficacy of such local treatments, especially for oligometastatic or oligoprogressive disease, has been documented [19, 34]. Combining modalities may further improve CR rates, thus expanding the utility of multidisciplinary approaches. In addition, uCN combined with ICI-based combination therapy was excluded from this study. Incorporating these strategies in future studies could reveal more therapeutic options. Finally, potential immortal time bias, particularly in relation to deferred cytoreductive nephrectomy, metastasectomy, and the response depth, could not be fully addressed. Therefore, these findings should be interpreted as descriptive rather than causal.

In conclusion, this study provides several important clinical messages in line with the proposed study framework. Approximately 25% of patients achieved medical CR or surgical CR following ICI-based combination therapy, indicating that complete disease eradication is achievable in select patients in real-world practice. Patients with medical CR and those with surgical CR had similarly favorable PFS and OS, thus supporting the clinical relevance of complete disease control regardless of the CR type. Surgical resection after systemic therapy may facilitate complete eradication of residual disease in select patients, even when medical CR is not achieved. Both deferred cytoreductive nephrectomy and metastasectomy were associated with similarly favorable outcomes. Finally, no significant differences between the IOIO and IOTKI groups regarding treatment CR, safety, or subsequent prognoses were observed, suggesting that both are effective options within a multidisciplinary treatment strategy. Prospective studies are warranted to define the optimal role and timing of multidisciplinary strategies involving ICI-based combination therapy.

## Supplementary Information

Below is the link to the electronic supplementary material.

Supplementary file1 (DOCX 20 KB)

## Data Availability

The datasets supporting the findings of this study are available from the corresponding author upon reasonable request. However, the data are written in Japanese and are subject to institutional regulations and privacy restrictions.

## Abbreviations

mRCC: Metastatic renal cell carcinoma
TKI: Tyrosine kinase inhibitor
ICI: Immune checkpoint inhibitor
CR: Complete response
OS: Overall survival
PFS: Progression-free survival
PR: Partial response
uCN: Upfront cytoreductive nephrectomy
dCN: Deferred cytoreductive nephrectomy
IOIO: ICI plus ICI
IOTKI: ICI plus TKI
IMDC: International metastatic RCC database consortium
TRAE: Treatment-related adverse event
ORR: Objective response rate
RFS: Recurrence-free survival
CT: Computed tomography
PD: Progressive disease
SBRT: Stereotactic body radiation therapy
