# Supplementary material for: Clinical evaluation of medical and surgical complete responses in metastatic renal cell carcinoma treated with immune checkpoint inhibitor combination therapy
Source: Int J Clin Oncol. 2026 Feb 6;31(4):683–94. doi: 10.1007/s10147-026-02981-9 (PMC13018086; doi:10.1007/s10147-026-02981-9)
Supplement: Supplementary file 1 — Supplementary file1 (DOCX 20 KB) [file 10147_2026_2981_MOESM1_ESM.docx]

**International Journal of Clinical Oncology**

**Clinical Evaluation of Medical and Surgical Complete Responses in Metastatic Renal Cell Carcinoma Treated with Immune Checkpoint Inhibitor Combination Therapy**

Kazuhiko Yoshida^a^, Tsunenori Kondo^b^, Junpei Iizuka^a^, Yuki Kobari^a^, Hiroki Ishihara^d^, Hironori Fukuda^a^, Hiroaki Shimmura^c^, Yasunobu Hashimoto^d^, Hiroshi Kobayashi^e^, Hideki Ishida^a^, Toshio Takagi^a^

Author’s Affiliations:

^a^ Department of Urology, Tokyo Women’s Medical University, 8-1, Kawada-cho, Shinjuku-ku, Tokyo 162-8666, Japan

^b^ Department of Urology, Tokyo Women’s Medical University, Adachi Medical Center, 4-33-1, Kouhoku, Adachi-ku, Tokyo, Japan

^c^ Department of Urology, Joban Hospital, Uenodai 57, Joban Kamiyunagayamachi, Iwaki, Fukushima, Japan

^d^ Department of Urology, Saiseikai Kawaguchi General Hospital, 5-11-5 Nishikawaguchi, Kawaguchi, Saitama, Japan

^e^ Department of Urology, Saiseikai Kazo Hospital, 1680 Kamitakayanagi, Kazo, Saitama, Japan

Corresponding author:

Kazuhiko Yoshida MD, PhD

Department of Urology, Tokyo Women’s Medical University, 8-1 Kawada-cho, Shinjuku Uku-ku, Tokyo 162-8666, Japan

E-mail: kzyoshida1@yahoo.co.jp

**Online Resource 1:** Efficacy of ICI-based combination therapy for mRCC according to complete response status and histopathological subtype

|  |  | Total | Medical  CR | Surgical CR | Non-CR | p-value |
| --- | --- | --- | --- | --- | --- | --- |
|  |  | n = 250 | n = 26 | n = 41 | n = 183 |  |
| Histopathology, n (%) | |  |  |  |  | 0.1884 |
|  | Clear cell RCC | 180 (72.0) | 23 (12.8) | 32 (17.8) | 125 (50.0) |  |
|  | Nonclear cell RCC | 43 (17.3) | 1 (0.4) | 5 (2.0) | 37 (14.8) |  |
|  | Unknown | 27 (10.8) | 2 (0.8) | 4 (1.6) | 21 (8.4) |  |

CR, complete response; ICI, immune checkpoint inhibitor; RCC, renal cell carcinoma.
